# Bias in bias recognition: People view others but not themselves as biased by preexisting beliefs and social stigmas

**DOI:** 10.1371/journal.pone.0240232

**Published:** 2020-10-09

**Authors:** Qi Wang, Hee Jin Jeon

**Affiliations:** 1 Department of Human Development, Cornell University, Ithaca, New York, United States of America; 2 Yonsei University, Seoul, South Korea; University of Vermont, UNITED STATES

## Abstract

Biases perpetuate when people think that they are innocent whereas others are guilty of biases. We examined whether people would detect biased thinking and behavior in others but not themselves as influenced by preexisting beliefs (myside bias) and social stigmas (social biases). The results of three large studies showed that, across demographic groups, participants attributed more biases to others than to themselves, and that this self-other asymmetry was particularly salient among those who hold strong beliefs about the existence of biases (Study 1 and Study 2). The self-other asymmetry in bias recognition dissipated when participants made simultaneous predictions about others’ and their own thoughts and behaviors (Study 3). People thus exhibit bias in bias recognition, and this metacognitive bias may be remedied when it is highlighted to people that we are all susceptible to biasing influences.

## Introduction

“Why do you look at the speck in your brother’s eye, but fail to notice the beam in your own eye?” (Matthew 7:3). As this biblical quotation suggests, people often recognize biases and flaws in others and yet overlook their own in everyday life. The double standard also frequently appears at the national stage, where conservatives and liberals frequently accuse each other of being irrational and biased while believing themselves to be rational and impartial [1–3]. Moreover, accusing one’s opponents of being “racists” or “sexists” has become commonplace in our increasingly polarized society [4]. The commitment to one’s own positions and righteousness and the condemnation of the opponents’ views and pitfalls have led to chaos interrupting healthy discourse between political parties and have challenged the intellectual openness on college campuses [1, 3, 5]. Given the critical real-life importance, the current research set out to empirically document the self-other asymmetry in people’s recognition of biased thinking and behavior that result from preexisting beliefs (i.e., myside bias) and social stigmas (i.e., social biases), and to further test the mechanism as well as remedy for this metacognitive bias in bias recognition.

Myside bias is a common type of cognitive bias where people process information in a manner biased toward their own prior beliefs, opinions, and attitudes [6–11]. It occurs when people seek, interpret, and remember evidence to confirm their preexisting beliefs and to refute opinions different from their own. For example, people with certain political affiliations may selectively tune in news channels that align with their own political views, take the broadcast information as truth, and remember the information over time in spite of new, view-opposing information from other sources. Some researchers consider myside bias to be a subclass of confirmation bias [12, 13], and some suggest that “myside bias” is a more precise term than “confirmation bias” to refer to the tendency of processing information to support one’s own position [7]. We thus use the term myside bias here. Myside bias reflects a deficiency in critical or rational thinking rather than general cognitive abilities or intelligence [7, 9, 13]. On the other hand, social biases, such as sexism, ageism, racism, and classism, are inclinations or prejudices against individuals or groups on the basis of gender, age, race, or social class.

Critically, myside bias and the various social biases can be possessed by both the self and others. Yet blaming others for these biases while believing oneself to be innocent is common in everyday life and has indeed become a major source of social-political tension in recent years [1, 3, 4]. This may reflect the general asymmetry in people’s perception of others and themselves documented in empirical research. It has been found that people tend to believe that, compared with others, they possess better attributes in various domains [14], have greater potential in the future [15], are less likely to experience negative events [16], and are more moral [17, 18] and less self-interested [19]. People also tend to believe that they see the world as it is whereas the divergent views of others reflect bias or a lack of objectivity [20, 21]. As a result, people exhibit a blind spot in their assessment of their own bias, viewing the judgments and behaviors of others as more susceptible to various cognitive and motivational biases than those of their own [17, 22, 23].

The current research is designed to investigate the asymmetry in people’s recognition of myside bias and social biases in others versus the self. It makes original contributions in several important ways. First of all, extant studies on the self-other asymmetry have mainly focused on cognitive biases involving self-enhancement or self-interest, whereby people more readily detect self-serving motives in others than in themselves [17, 19, 22]. We focused on the self-other asymmetry in the recognition of myside bias, examining the extent to which people view others’, but not their own, thinking and behavior as being influenced by preexisting beliefs and attitudes–biasing influences that may be subtle and yet have important social and political consequences [1, 3, 5]. We further examined the self-other asymmetry in the recognition of various social biases—sexism, ageism, racism, and classism, which, to our knowledge, has not been empirically documented and yet can have the most detrimental consequence for the society [4].

Second, rather than studying college students’ judgements about controversial or politically charged issues as many prior studies have done, we focused on judgments in everyday situations. In so doing, we aimed to eliminate the potential interference of personal ideologies and also to obtain generalizable results beyond specific issues. Furthermore, we recruited large and diverse samples and examined the influence of demographic factors, including age, gender, ethnicity, and education, on the recognition of myside bias and social biases and the associated self-other asymmetry in everyday contexts. In spite of their importance, demographical factors have not been considered in previous studies in this area and have been much neglected in psychological research [24–26].

Third, in previous studies of perceptions of bias in self versus others, participants were typically asked to estimate the degree to which the judgments of others or their own might be influenced by bias, or to make explicit self-to-other comparisons on susceptibility to bias [22, 23]. This direct approach may activate social desirability concerns and self-serving motives, which can then lead participants to attribute less bias to themselves than to others [27]. We used a more subtle or indirect approach in the current studies. Participants were asked to predict others’ or their own thoughts, feelings, and behaviors in specific situations where one might be influenced by prior beliefs or social stigma. The self-other asymmetry in bias recognition would occur when participants believe that prior beliefs or social stigma play a considerable role in determining others’ thoughts, feelings, and behaviors and yet matter little for themselves.

Fourth, we tested a mechanism underlying the self-other asymmetry in bias recognition, namely, people’s explicit beliefs about the existence of myside bias or social biases. Although the cognitive processes implicated in people’s biased judgements may be nonconscious, explicit beliefs about a bias may guide people’s decision making in attributing the bias to others but not themselves. As a result, people who hold stronger beliefs about the prevalence of a bias may exhibit greater self-other asymmetry in bias recognition. To our knowledge, there has been little to none empirical work to directly test the connection between unconscious or implicit biased reasoning and explicit beliefs about the relevant biases [21]. We tested this connection in the context of the recognition of myside bias and social biases in self versus others.

Finally, we explored a potential remedy for the bias in bias recognition, which has not been attempted in prior research. The self-other asymmetry in bias recognition may reflect either a heightened sensitivity to bias in others, or an attenuated sensitivity or “blindness” to bias in oneself, or both. Research on theory of mind and social perspective-taking has revealed the ability among individuals to identify with other people and see things from others’ perspectives in social situations [28–30]. We thus raised participants’ “social mindfulness” by asking them to judge side-by-side others’ and their own thoughts, feelings, and behaviors in the same situations where myside bias might occur. The self-other asymmetry would lessen or dissipate when participants attribute less bias to others, more bias to themselves, or both, when compared with a control condition.

In sum, we investigated the asymmetry in people’s recognition of myside bias and social biases in others’ versus their own thoughts, feelings, and behaviors in everyday contexts in large and diverse samples. We predicted that 1) people would attribute more myside bias (Study 1) and social biases (Study 2) to others than to themselves; 2) people with stronger explicit beliefs about myside bias (Study 1) or social biases (Study 2) would show greater self-other asymmetry in bias recognition; and 3) people would show reduced self-other asymmetry in bias recognition when making simultaneous judgments about themselves and others (Study 3). We tested the effects of demographic factors on bias recognition, although we made no a priori predictions given the lack of relevant data in prior research.

### Ethics statement

All data and test materials are available in Supporting Information. Test materials are also available at https://dx.doi.org/10.17504/protocols.io.bjiikkce. This research was approved by Cornell University Institutional Review Board (IRB) for Human Participants (Protocol ID#: 1705007173). Participants indicated their agreement to participate in a written consent at the initiation of the studies.

## Study 1: Bias in recognizing myside bias

Myside bias is a common product of human cognitive irrationality that occurs when people fail to decouple their prior beliefs and opinions from the evaluation of a current situation [7, 9, 11]. Do people recognize this failure equally in others and themselves? We tested in this study the self-other asymmetry in myside bias recognition and also the potential effect of demographic factors on the asymmetry. Furthermore, we examined the role of explicit beliefs about myside bias in moderating the asymmetry. Although people may consciously recognize the existence of particular biases and may not view themselves as immune to such biases on abstract terms, they may be inclined to think that they are innocent whereas others are guilty of the biases in the assessment of specific situations [20–22]. We predicted that participants would regard others as more likely than themselves to be influenced by preexisting beliefs and attitudes. We further predicted that the self-other asymmetry in myside bias recognition would be particularly salient among those who hold strong beliefs about myside bias.

### Method

#### Participants

Participants (*N* = 1,014) aged 18 to 74 years (*M* = 35.65, *SD* = 11.31, *median* = 32.33) were recruited through Amazon’s Mechanical Turk [MTurk; 31]. To obtain high-quality data, the approval rate was set at 95% (i.e., at least 95% of the participants’ previous tasks on MTurk were approved by other researchers), the Number of HITs Approved was set at 1000 (i.e., the participants had successfully completed at least 1000 MTurk studies), and blocking duplicate IDs was set to ensure that the same participants would not take the survey multiple times [32]. Each participant received $1.00 for taking part in the study. Additional demographic information is reported in Table 1.

**Table 1:** Demographic variables and means and standard deviations of bias recognition scores.

	Demographic variable			Other condition			Self condition	
			n	Mean	SD	n	Mean	SD
**Study 1**^a^	**Age (medium split)**	**Old**	245	0.40	0.66	257	0.12	0.45
		**Young**	257	0.13	0.63	250	-0.08	0.45
	**Gender**	**Female**	236	0.32	0.68	245	0.05	0.45
		**Male**	263	0.20	0.63	259	0.00	0.48
	**Ethnicity**	**African**	30	0.37	0.65	39	0.07	0.44
		**Asian**	111	-0.04	0.62	117	-0.22	0.44
		**Caucasian**	312	0.39	0.64	295	0.10	0.43
		**Hispanic**	29	0.22	0.61	30	0.19	0.50
	**Education**	**Graduate**	173	0.07	0.71	171	-0.16	0.44
		**College**	229	0.36	0.60	225	0.08	0.45
		**High school**	94	0.38	0.60	103	0.19	0.44
**Study 2**^b^	**Age (medium split)**	**Old**	237	16.42	15.62	260	4.19	14.21
		**Young**	263	11.02	15.03	243	4.47	11.41
	**Gender**	**Female**	259	14.31	16.11	227	3.30	11.91
		**Male**	236	12.53	14.79	277	5.14	13.65
	**Ethnicity**	**African**	29	13.71	15.03	35	6.30	16.30
		**Asian**	146	9.07	14.53	136	2.50	9.44
		**Caucasian**	288	16.33	16.08	292	5.03	14.14
		**Hispanic**	22	10.61	10.76	20	4.70	10.32
	**Education**	**Graduate**	167	10.25	13.45	196	2.74	10.42
		**College**	233	15.09	16.77	210	5.95	13.04
		**High school**	95	15.62	15.05	87	3.37	16.79
**Study 3**^c^	**Age (medium split)**	**Old**	253	1.68	0.88	229	1.29	0.84
		**Young**	246	1.26	1.12	279	0.96	0.94
	**Gender**	**Female**	230	1.60	1.00	222	1.22	0.89
		**Male**	269	1.35	1.04	285	1.02	0.92
	**Ethnicity**	**African**	30	1.56	1.16	27	0.89	1.03
		**Asian**	106	1.16	1.06	113	0.79	0.93
		**Caucasian**	313	1.58	0.96	326	1.22	0.89
		**Hispanic**	25	1.63	1.07	27	1.31	0.65
	**Education**	**Graduate**	145	1.27	1.00	169	0.89	0.91
		**College**	261	1.53	1.02	241	1.15	0.89
		**High school**	89	1.61	1.08	93	1.37	0.87

The self-other asymmetry in bias recognition was evident across demographic groups.

^**a**^ In Study 1, analyses involving demographic factors excluded 5 participants who did not provide age information, 11 participants who reported being both male and female or transgender or who did not provide gender information; 19 participants who reported atypical types of education or did not report the information; and 12 native Americans, 38 from other racial groups, and 1 with no ethnicity information.

^**b**^ In Study 2, the average social bias recognition score across the four social biases is presented. Analyses involving demographical factors excluded 1 participant who did not provide age information; 5 participants who reported being both male and female or transgender or who did not provide gender information; 16 participants who reported atypical types of education or did not report the information; and 15 native Americans and 21 from other racial groups.

^**c**^ In Study 3, the myside bias recognition score for Self vs. Other 1 is presented. Analyses involving demographical factors excluded 2 participants who did not provide age information; 3 participants who did not provide gender information; 11 participants who reported atypical types of education or did not report the information; and 9 native Americans, 31 from other racial groups, and 2 with no ethnicity information.

#### Measures and procedure

Participants were randomly assigned to either the self (*n* = 509) or other (*n* = 505) condition. They were presented with 16 hypothetical scenarios and were asked to predict their own or another person’s thoughts (e.g., to think of a $50 necklace as fake or real), feelings (e.g., to feel about a party as boring or fun), and behaviors (e.g., to buy Adidas or Nike soccer cleats) that could be influenced by prior beliefs, opinions, and attitudes (See Table 2 for the scenario themes). For example:

“You are (Nancy is) a professor evaluating a job candidate, Bill, for your (her) department. You (Nancy) know(s) Bill personally and really like(s) him. Bill has published on average 3 articles a year, which is about the same as the average number of publications by the faculty in the department. How would you (Nancy) evaluate Bill’s performance?”

**Table 2:** Themes of the hypothetical scenarios.

**Study 1**		
**Dress**	Theme: You (Jessica) heard that Princess M’s dress was white.	
	How likely to remember Princess M’s whitish-pinkish dress as white/pink?	
**Party**	**Theme:** You (John) think(s) that Eric is a boring person.	
	How likely to feel about Eric’s party as boring/fun?	
**Soccer Cleats**	**Theme:** You (Jack) saw your (his) least favorite team wear Adidas shorts.	
	How likely to buy Adidas/Nike soccer cleats?	
**Professor**	**Theme:** You (Nancy) know(s) Bill personally and really like(s) him.	
	How likely to evaluate Bill’s performance as unproductive/productive?	
**Lunch**	**Theme:** You (Janice) heard that Koko’s budae-jjigae was overpriced and salty.	
	How likely to feel about Koko’s budae-jjigae as terrible/delicious?	
**Class**	**Theme:** You (Erica) heard good reviews about a class.	
	How likely to rate the class as poor/excellent?	
**Movie**	**Theme:** You (Justin) dislike(s) musicals but enjoyed La La Land.	
	How likely to rate La La Land for 1 star/5 stars?	
**Donation**	**Theme:** You (Steve) dislike(s) Sarah and think(s) she is mean.	
	How likely to judge Sarah’s donation as very meager/very generous?	
**Ucro Necklace**	**Theme:** You (Sally) know(s) Ucro is a precious metal for high-end jewelry.	
	How likely to think of a $50 Ucro necklace as fake/real?	
**Ice hockey**	**Theme:** You (Sylvia) rooted for the home team at a hockey game that tied.	
	How likely to evaluate the team’s performance as poor/great?	
**Anker Spearhead**	**Theme:** You (Brandon) learned that Anker is an ancient bronze spearhead.	
	How likely to judge the claim of finding Anker on farmland as reliable/unreliable?	
**Dooyu Soymilk**		**Theme:** You (Jacob) endorse(s) a soymilk company Dooyu, not its rival Rego. How likely to prefer Dooyu/Rego soymilk?	
**Apples**	**Theme:** You (Casey) distrust(s) the marketing scheme to sell apples.	
	How likely to follow the recommendation of a health report to eat apples?	
**ASO computers**	**Theme:** You (Sam) plan(s) to buy a supercomputer but it has bad reviews.	
	How likely to buy/not buy the supercomputer?	
**Books**	**Theme:** You (Carol) think(s) fiction books are boring but enjoyed one lately.	
	How likely to remember the book as boring/fascinating?	
**Babysitter**		**Theme:** You (Ode) are (is) hiring a babysitter: Jenny is recommended by friends and Tina is not. How likely to choose Jenny/Tina as the babysitter?	
**Study 2**		
**Sexism**	Lecturer	Hiring Sara vs. Chris as lecturer
	Soccer Team	Funding the Men’s vs. the Women’s soccer team
	Fellowship	Recommending Jane vs. Adam for a fellowship
	Neurologist	Choosing Tom vs. Emily as neurologist
**Ageism**	Programmer	Recruiting Adam (age 25) vs. Seth (age 55) as programmer
	Dancer	Choosing Linsey (age 38) vs. Sarah (age 21) as lead dancer
	Family restaurant server	Hiring Michael (age 27) vs. Ross (age 53) as server
	Attorney	Choosing Bridget (age 65) vs. Melissa (age 42) as attorney
**Racism**	Senior Associate Dean	Hiring Juan Martinez vs. William Silbey as associate dean
	Limo driver	Choosing Kelly Smith vs. Lakisha Lacks as limo driver
	Dental assistant	Hiring Ximena Washington vs. Ashely Johnson as assistant
	Sous Chef	Hiring Billy Brown vs. Mateo Begay as new sous chef
**Classism**	Tenant	Choosing John (professor) vs. Max (plumber) as tenant
	Restaurant seating	Seating a construction worker vs. a lawyer first
	Tennis team captain	Selecting Lisa (lower class) vs. Jane (upper class) as captain
	Babysitter	Hiring Jed (college graduate) vs. Tim (drop-out) as babysitter
**Study 3**		
**Dress**	Theme: You (Jessica) heard that Princess M’s dress was white while Emily heard that it was pink.	
	How likely to remember Princess M’s whitish-pinkish dress as white/pink?	
**Party**	**Theme:** You (John) think(s) that Eric is a fun person while Adam thinks he is boring.	
	How likely to feel about Eric’s party as boring/fun?	
**Soccer Cleats**	**Theme:** You (Jack) saw your least favorite team wear Adidas shorts while Nick saw a cool Adidas T-shirt commercial.	
	How likely to buy Adidas/Nike soccer cleats?	
**Professor**	**Theme:** You (Nancy) know(s) Bill personally and like(s) him while Linda does not think highly of his work.	
	How likely to evaluate Bill’s performance as unproductive/productive?	
**Lunch**	**Theme:** You (Janice) heard that Koko’s budae-jjigae was overpriced and salty while Judy heard it was delicious.	
	How likely to feel about Koko’s budae-jjigae as terrible/delicious?	
**Class**	**Theme:** You (Erica) heard good reviews about a class while Dara heard bad reviews about it.	
	How likely to rate the class as poor/excellent?	
**Movie**	**Theme:** You (Justin) love(s) musicals while Brian hates them.	
	How likely to rate La La Land for 1 star/5 stars?	
**Donation**	**Theme:** You (Steve) dislike(s) Sarah while Bob likes her.	
	How likely to judge Sarah’s donation as very meager/very generous?	

Here, the self-other asymmetry in myside bias recognition would occur when participants predicted that the personal fondness for Bill would lead to a more favorable evaluation of Bill’s performance by Nancy (other condition) than by themselves (self condition). Participants were asked to indicate on 9-point scales their predictions (e.g., from unproductive (-4) to somewhat in between (0) to productive (+4), for the above example scenario). Negatively worded scenarios were reverse scored, so that positive values indicated recognition of myside bias where participants predicted the thoughts of the self or others (e.g., +4: “Bill is productive”) in line with prior beliefs and attitudes (e.g., “I [Nancy] like[s] Bill”). A myside bias recognition score was then calculated by averaging the ratings across the 16 scenarios, which reflected the extent to which participants attributed myside bias to others (in the other condition) or themselves (in the self condition).

We developed a Beliefs about Myside Bias (BMB) scale to measure participants’ explicit beliefs about myside bias. The scale consisted of 10 statements that described self-confirmation tendencies in the general population (e.g., “People see what they want to see,” “People find excuses to refute a position they disagree with,” and reverse scored items, e.g., “People are open to views different from their own”). Participants indicated their responses on 11-point scales ranging from strongly disagree (0) to strongly agree (10). The scale measure showed satisfactory internal consistency, with a Cronbach’s α = .77. A total score was summed across the statements (maximum = 100) to index beliefs about myside bias.

### Results

To test the self-other asymmetry, an independent-samples t-test with condition as the independent variable was first conducted on the bias recognition score. The analysis revealed that participants attributed greater myside bias to others (*M* = 0.26, *SD* = 0.66) than to themselves (*M* = 0.02, *SD* = 0.46), *t*(1009) = 6.76, *p* < .0001, *d* = .43.

Then, to test the possible influence of demographic factors on the self-other asymmetry, a regression analysis was conducted with condition, demographic variables (i.e., age, gender, ethnicity, and education), and the interactions between condition and demographic variables as predictors. The condition effect remained significant, *t* = 3.49, *B* = .09, *p* = .0005. No significant interaction between condition and demographic variables was observed. Thus, the self-other asymmetry in myside bias recognition was evident across demographic groups (Table 1). In addition, a significant main effect of age emerged, *t* = 4.17, *B* = .08, *p* < .0001, whereby older participants in both conditions perceived greater myside bias than did younger participants. There were also significant main effects of ethnicity, *F*(3, 912) = 7.83, *p* < .0001, and education, *F*(2, 912) = 8.89, *p* = .0002. Subsequent Tukey HSD tests (p < .05) showed that across conditions Caucasian and African American participants perceived greater myside bias than did Asians, whereas Hispanic participants did not differ significantly from any group. Participants with graduate degrees perceived less myside bias than did those with high-school and college degrees, who did not significantly differ from each other.

**Fig 1:**
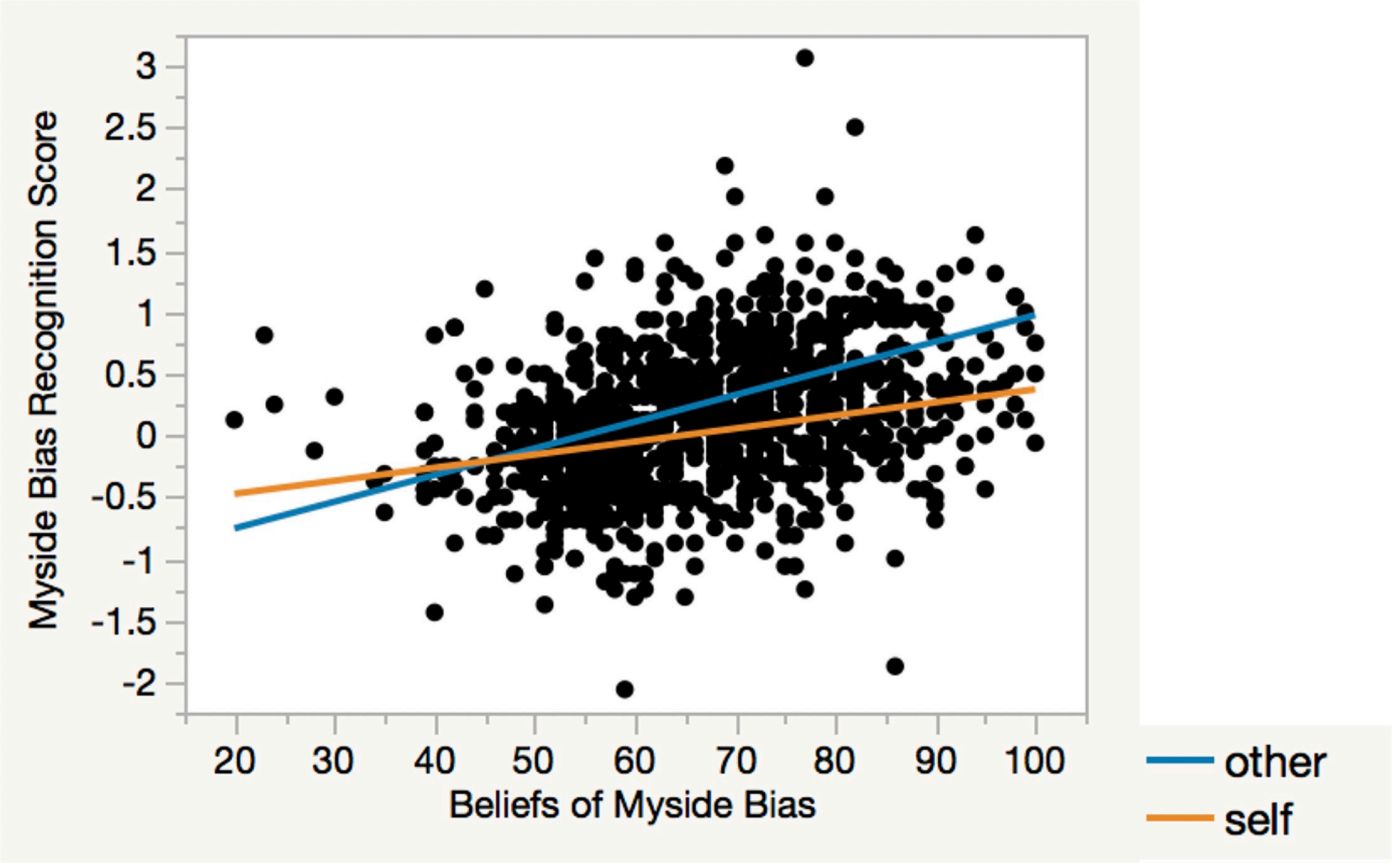
Myside bias recognition in self and others as a function of beliefs about myside bias. The self-other asymmetry in myside bias recognition was greater among those with stronger beliefs about myside bias.

To examine the role of explicit beliefs about myside bias in moderating the self-other asymmetry, a regression analysis was conducted on the bias recognition score, with condition, BMB, and a condition x BMB interaction as predictors. The analysis yielded main effects of condition, *t* = 7.24, *B* = .12, *p* < .0001, and BMB, *t* = 13.07, *B* = .02, *p* < .0001, and a Condition x BMB interaction, *t* = 4.47, *B* = .01, *p* < .0001. As predicted, the self-other asymmetry in myside bias recognition was greater among participants with stronger beliefs about myside bias (Fig 1).

In summary, across demographic groups, participants attributed greater myside bias to others than to themselves. This self-other asymmetry was further moderated by explicit beliefs about myside bias, such that participants who held stronger beliefs attributed greater myside bias to others relative to themselves than those with weaker beliefs. Thus, although people may believe the prevalence of myside bias, they are inclined to apply the bias to others’, but not their own, thoughts, feelings, and behaviors in everyday settings. In addition, older, Caucasian and African American, and high-school- and college-degree participants perceived greater myside bias in both self and others than did younger, Asian, and graduate-degree participants, respectively.

## Study 2: Bias in recognizing social biases

We tested in this study the self-other asymmetry in the recognition of social biases and the moderation role of explicit beliefs about social biases in bias recognition. We predicted that participants would readily attribute social biases, including sexism, ageism, racism, and classism, to others rather than themselves in everyday situations. Furthermore, we predicted that those who hold stronger beliefs about the existence of social biases would exhibit a greater self-other asymmetry in bias recognition. In addition, given the findings from Study 1, we expected that the self-other asymmetry in the recognition of social biases would be evident across demographic groups.

### Method

#### Participants

A new group of participants (*N* = 1,004) who did not partake in Study 1 was recruited through MTurk. Their age ranged from 18 to 80 years (*M* = 35.75, *SD* = 11.68, *median* = 32.00). The same participant selection criteria were used as in Study 1. Each participant received $1.00 for taking part in the study. Additional demographic information is reported in Table 1.

#### Measures and procedure

Participants were randomly assigned to either the self (*n* = 504) or other condition (*n* = 500). They were presented with 16 hypothetical scenarios, with 4 for each type of bias: sexism, ageism, racism, and classism. Participants were asked to predict their own or another person’s behaviors or choices that were likely to be influenced by social biases (See Table 2 for the scenario themes). For example:

“You are (Sterling is) looking around for a neurologist for an aging parent. Two doctors are highly recommended by others, Tom and Emily. Which doctor are you (is Sterling) likely to choose?”

Here, the self-other asymmetry in social bias (i.e., sexism) recognition would occur when participants predicted others (other condition) to be more likely than themselves (self condition) to choose Tom over Emily. Participants were asked to assign a total 100% between a choice that reflected a social bias (e.g., Tom, where a man was selected over an equally qualified woman) and a choice that countered the social bias (e.g., Emily). The order in which the choices were presented was counterbalanced across scenarios, such that for each social bias, two scenarios listed the bias choice first and two listed the counter-bias choice first. In addition, for each scenario, half of the participants assigned a percentage to the bias choice first and the other half assigned a percentage to the counter-bias choice first. The average percentages assigned to the bias and counter-bias choices across the 4 scenarios were calculated for each social bias.

We developed a Beliefs about Social Biases (BSB) scale to measure participants’ explicit beliefs about social biases, which included four statements concerning social biases in the general population: “Many people are sexists,” “People are generally ageists,” “Most people have the tendency of being racists,” and “People are often biased against those from lower socio-economic status.” Participants indicated their responses on 11-point scales from strongly disagree (0) to strongly agree (10). Ratings on the four biases showed high internal consistency, with a Cronbach’s α = .85. The BSB rating score for each social bias was submitted to respective analyses.

### Results

**Fig 2:**
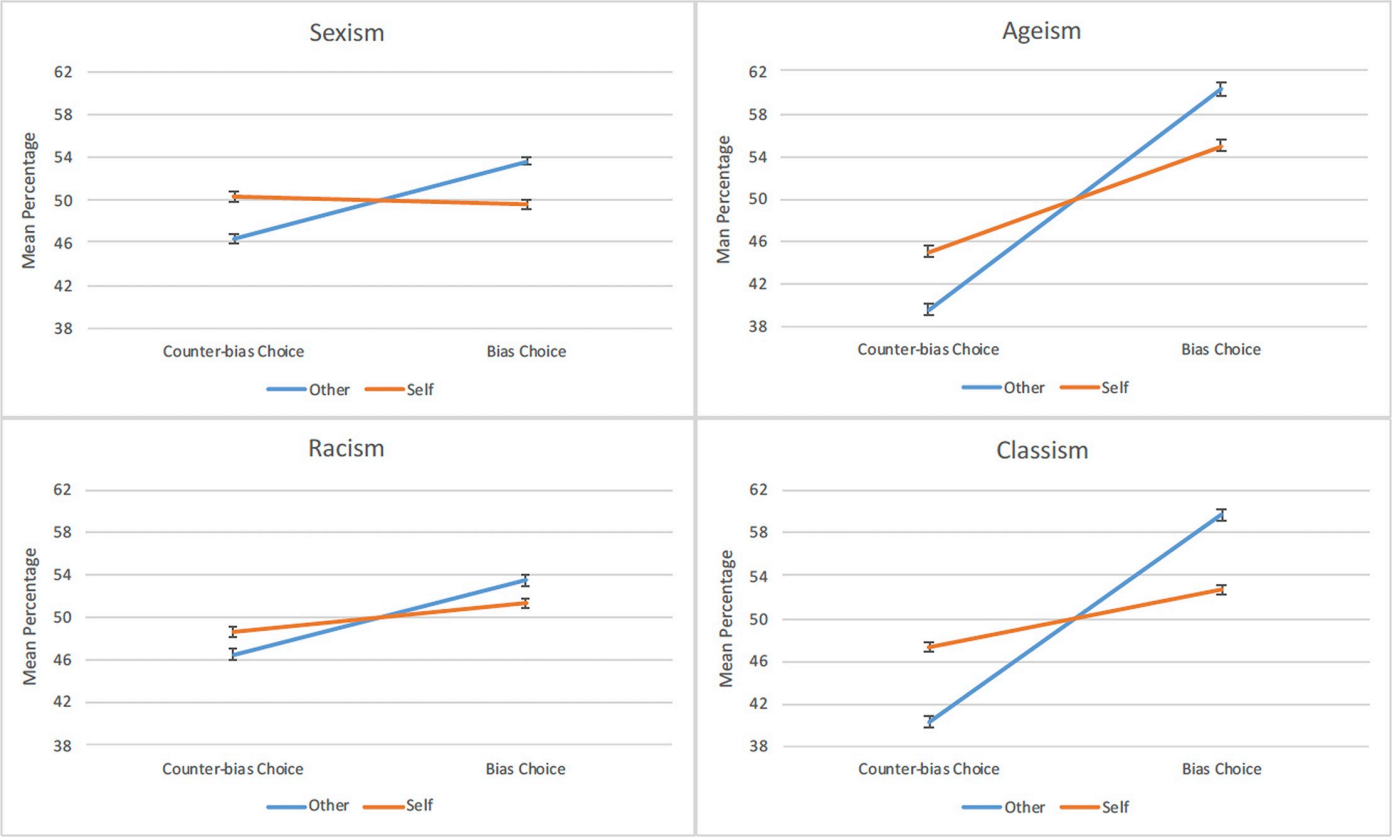
Social bias recognition in self and others. Across all types of social biases, participants attributed more biases to others than to themselves. Error bars represent standard errors of the mean.

We first tested the self-other asymmetry in the recognition of social biases. Note that the percentages assigned to the bias and counter-bias choices summed to 100%. Thus, for each social bias, only the percentage assigned to the bias choices was submitted to an independent-samples t-test with condition as the independent variable. Across all social biases, participants in the other condition assigned a greater percentage to the bias choices than did those in the self condition, sexism *t*(1002) = 6.89, *p* < .0001, *d* = .44; ageism *t*(1002) = 6.82, *p* < .0001, *d* = .43; racism *t*(1002) = 3.17, *p* = .0016, *d* = .20; classism *t*(1002) = 9.53, *p* < .0001, *d* = .60. This pattern of results was reversed for the counter-bias choices. In other words, participants consistently viewed others as more likely to make choices in line with social biases and less likely to make counter-bias choices than themselves (Fig 2).

**Fig 3:**
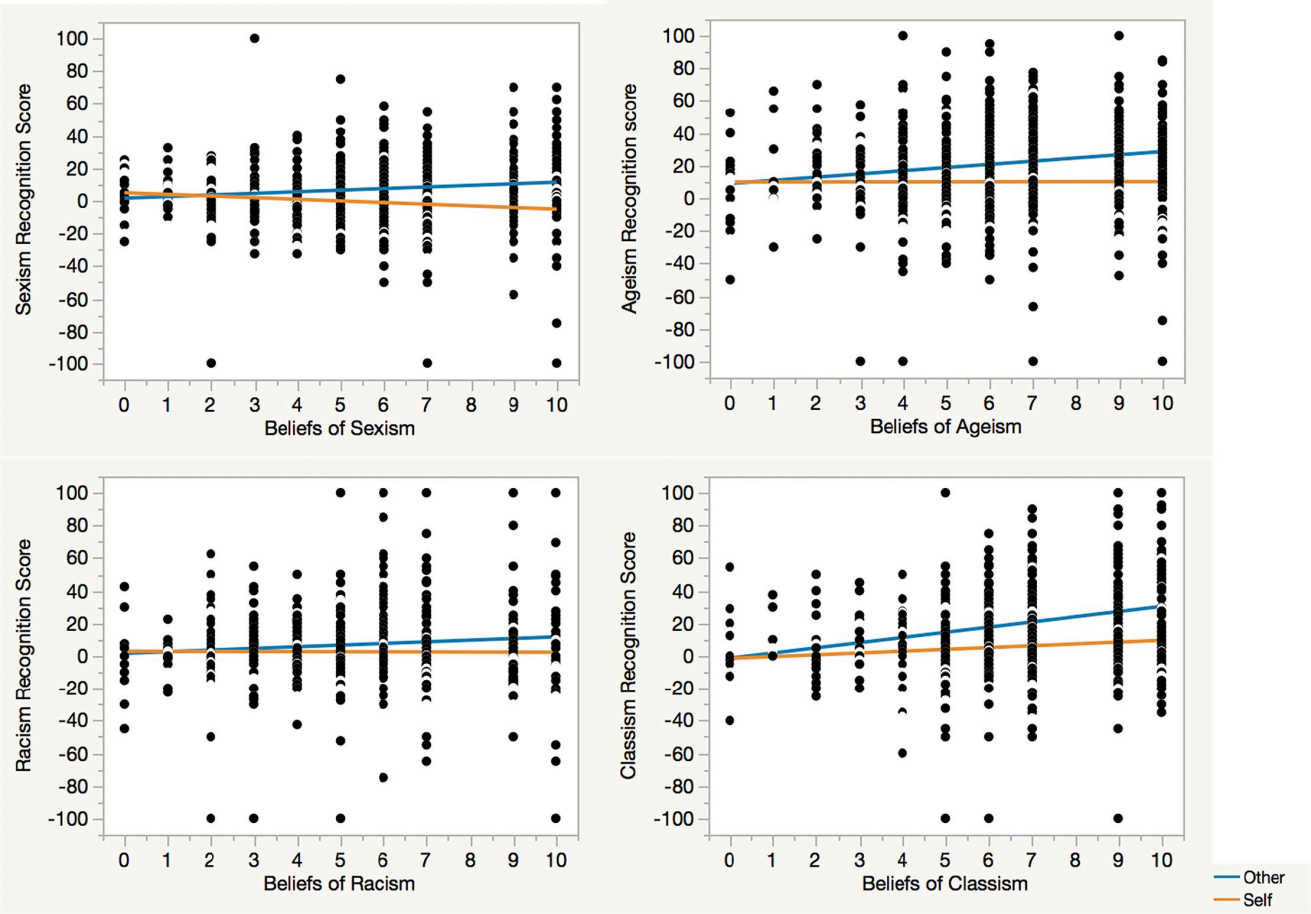
Social bias recognition in self and others as a function of beliefs about social biases. Across all types of social biases, the self-other asymmetry in bias recognition was greater among those with stronger beliefs about social biases.

For subsequent analyses that examined the moderation role of explicit beliefs about social biases in bias recognition, a composite bias recognition score was calculated for each social bias by subtracting the average percentage assigned to the counter-bias choices from the average percentage assigned to the bias choices, with higher scores indicating greater social bias recognition. A regression analysis was conducted on each social bias recognition score, with condition, BSB, and a condition x BSB interaction as predictors. The analyses consistently revealed a main effect of condition, sexism *t* = 6.92, *B* = 3.99, *p* < .0001; ageism *t* = 6.52, *B* = 5.16, *p* < .0001; racism *t* = 3.15, *B* = 2.11, *p* = .0017; classism *t* = 9.32, *B* = 6.65, *p* < .0001. There was also a main effect of BSB except for sexism, sexism *t* = -.05, *B* = -.01, *p* = .96; ageism *t* = 3.22, *B* = 1.00, *p* = .0013; racism *t* = 1.88, *B* = .48, *p* = .061; classism *t* = 7.33, *B* = 2.15, *p* < .0001. Most important, a Condition x BSB interaction emerged across all social biases, sexism *t* = 4.53, *B* = 1.00, *p* < .0001; ageism *t* = 3.12, *B* = .96, *p* = .0019; racism *t* = 2.11, *B* = .54, *p* = .035; classism *t* = 3.53, *B* = 1.03, *p* = .0004. Thus, as predicted, participants with stronger beliefs about social biases exhibited a greater self-other asymmetry in bias recognition (Fig 3).

Finally, to test the potential effect of demographic variables on the self-other asymmetry across social biases, a mean social bias recognition score was calculated by averaging the composite bias recognition scores across the four social biases (Table 1). The mean score was then submitted to a regression analysis, with condition, demographic variables, and the interactions between condition and demographic variables as predictors. The condition effect remained significant, *t* = 5.46, *B* = 4.24, *p* < .0001. There was only an interaction between condition and age, *t* = 2.25, *B* = 1.08, *p* = .025, whereby older participants (LSMeans difference = 12.23, *p* < .05) exhibited a greater self-other asymmetry than did younger participants (LSMeans difference = 6.54, *p* < .05). The self-other asymmetry in social bias recognition was thus evident across demographic groups. In addition, a main effect of ethnicity emerged, *F*(3, 932) = 2.61, *p* = .050. Subsequent Tukey HSD tests (p < .05) showed that across conditions Caucasian participants perceived greater social biases than did Asians, while African American and Hispanic participants fell in between and did not significantly differ from any group.

In summary, across different social biases and different demographic groups, participants attributed greater biases to others than to themselves. This self-other asymmetry was further moderated by explicit beliefs about social biases, such that those with stronger beliefs exhibited a greater self-other asymmetry in social bias recognition. Thus, although people may strongly believe the wide existence of social biases, they apply the biases only to others but view themselves as immune to the biases in everyday settings. In addition, the self-other asymmetry in social bias recognition was stronger among older than younger participants, and Caucasian participants perceived greater social biases in both themselves and others than did Asians.

## Study 3: Reducing self-other asymmetry in bias recognition

Studies 1 and 2 consistently demonstrated a self-other asymmetry in bias recognition. Is it possible to reduce or even overcome this metacognitive bias? In the first two studies, as in most studies of self-other comparisons [15, 17, 22, 23], participants were asked to make judgments of either others’ or their own thoughts and behaviors. Research on theory of mind and social perspective-taking has shown that people often come to sympathize with others and their perspectives in social situations [28–30]. Thus, when given the opportunity to make predictions about others alongside with themselves, people may be more mindful of the cognitive processes that equally underlie their own and others’ thoughts and behaviors and, consequently, overcome the self-other asymmetry in bias recognition. Study 3 tested this possibility by asking participants to predict both their own and others’ thoughts, feelings, and behaviors that could be influenced by myside bias, in comparison with a control group in which participants made predictions only about others. We predicted that the self-other asymmetry in myside bias recognition would dissipate when participants made predictions about self and other side-by-side, and that these participants would also attribute less myside bias overall to themselves and others than those who made predictions only about others. Note that we did not explicitly ask participants to make direct self-to-other comparisons, so as to avoid possible influence of social desirability concerns and self-serving motives [27].

### Method

#### Participants

A new sample of participants (*N* = 1,009) aged 17 to 77 years (*M* = 35.41, *SD* = 11.20, *median* = 33.00) was recruited through MTurk. The same participant selection criteria were used as in Study 1 and Study 2. Each participant received $1.00 for taking part in the study. Additional demographic information is reported in Table 1.

#### Measures and procedure

Participants were randomly assigned to a self-other (*n* = 508) or other-other condition (*n* = 501). They were presented with 8 hypothetical scenarios and were asked to predict their own and another person’s (i.e., “Self” and “Other^2^” in the self-other condition) or two other people’s (i.e., “Other^1^” and “Other^2^” in the other-other condition) thoughts, feelings, and behaviors that could be influenced by prior beliefs, attitudes, and opinions. Across the scenarios, Self in the self-other condition and Other^1^ in the other-other condition played identical roles, whereas Other^2^ in the two conditions played identical roles. In each scenario, Self (Other^1^) and Other^2^ were described as having different preexisting beliefs or attitudes (See Table 2 for the scenario themes). For example:

“You (Steve), Bob, and Sarah work in the same company. You (Steve) dislike(s) Sarah and think(s) she is mean. Bob likes Sarah and thinks she is nice. People in the company were asked to donate money for a colleague whose house was recently burned down. Sarah put in $50. What would you (Steve) and Bob think about the amount of Sarah’s donation?”

Here, in line with the hypothesis, participants in the self-other condition would predict that they and Bob were to think of similarly Sarah’s donation in spite of their different prior attitudes toward Sarah. They would also perceive their and Bob’s prior attitudes as less influential on their thoughts of Sarah’s donation than would participants in the other-other condition who made predictions about Steve’s and Bob’s thoughts. In other words, participants in the self-other condition would attribute a similar magnitude of myside bias to themselves and Bob, and they would also attribute less myside bias to both themselves and Bob than those in the other-other condition in attributing myside bias to Steve and Bob.

For both Self (Other^1^) and Other^2^ (e.g., you [Steve] and Bob), participants were asked to indicate on 9-point scales their predictions (e.g., from very meager [–4] to somewhere in between [0] to very generous [+4], for the above example scenario). The order in which participants made predictions about Self (Other^1^) and Other^2^ was counterbalanced such that for each scenario, half of the participants made the prediction about Self (Other^1^) first and the other half made the prediction about Other^2^ first. Negatively worded scenarios were later reverse scored, so that positive values indicated recognition of myside bias where participants predicted the thoughts of the self or others (e.g., +4: “Sarah’s donation is very generous”) in line with prior beliefs and attitudes (e.g., “Sarah is nice”). Participants each received two myside bias recognition scores for their attribution of bias to Self (Other^1^) and Other^2^, with the respective ratings averaged across the scenarios.

### Results

**Fig 4:**
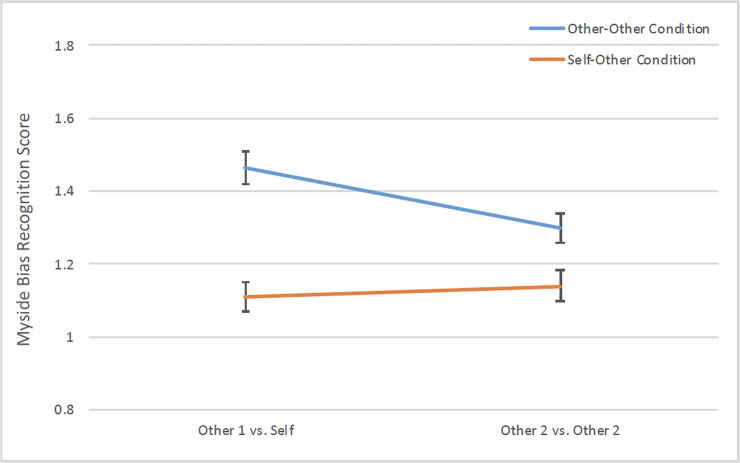
Myside bias recognition in self and others as a function of condition. The self-other asymmetry dissipated in the self-other condition (orange line) whereby participants recognized similar amount of myside bias in self and other^2^ and they also attributed less bias overall than did those in the other-other condition (blue line), error bars represent standard errors of the mean.

To examine the recognition of myside bias within and between conditions, a 2 (condition: self-other vs. other-other) x 2 (person: Self/Other^1^ vs. Other^2^) mixed-model ANOVA on the myside bias recognition score was conducted. The analysis yielded main effects of condition, *F*(1, 1003) = 21.42, *p* < .0001, *η*_*p*_*2* = .021, and person, *F*(1, 1003) = 8.60, p = .0034, *η*_*p*_*2* = .009, qualified by a Condition x Person interaction, *F*(1, 1003) = 17.64, *p* < .0001, *η*_*p*_*2* = .017 (Fig 4). As predicted, the self-other asymmetry dissipated in the self-other condition such that there was no significant difference in bias recognition between Self (*M* = 1.11, *SD* = .91, 95% CI = [1.03, 1.19]) and Other^2^ (*M* = 1.14, *SD* = .95, 95% CI = [1.06, 1.22]), *F*(1, 505) = .79, *p* = .38, *η*_*p*_*2* = .002, whereas participants in the other-other condition attributed more bias to Other^1^ (*M* = 1.46, *SD* = 1.03, 95% CI = [1.37, 1.55]) than Other^2^ (*M* = 1.30, *SD* = .95, 95% CI = [1.22, 1.38]), *F*(1, 498) = 25.96, *p* < .0001, η_p_2 = .050. When compared between conditions, participants in the self-other condition attributed less bias overall than those in the other-other condition, and the tendency to attribute less bias was stronger for the self (i.e., Self vs. Other^1^), *t*(1005) = 5.83, *p* < .0001, *d* = .37, than for others (i.e., Other^2^ vs. Other^2^), *t*(1004) = 2.68, *p* = .0074, *d* = .17. These results suggest that the diminished self-other asymmetry in the self-other condition was due to a decreased bias attribution to others rather than an increased bias attribution to the self.

To replicate the between-condition self-other asymmetry in myside bias recognition across demographic groups, a regression analysis was conducted on the bias recognition score for Self vs. Other^1^, with condition, demographic variables, and their interactions as predictors. The results showed that, consistent with the findings of Study 1, the between-condition difference in myside bias recognition for Self vs. Other^1^ was evident across demographic groups, *t* = 4.14, *B* = .21, *p* < .0001, and that there was no significant interaction between condition and demographic variables (Table 1). In addition, across conditions, older participants perceived greater myside bias than did younger participants, *t* = 4.85, *B* = .15, *p* < .0001, and females perceived greater myside bias than did males, *t* = 2.38, *B* = .07, *p* = .017. A significant main effect of ethnicity also emerged, *F*(3, 940) = 3.42, *p* = .017. Caucasian participants perceived greater myside bias than did Asians, and Hispanic and African American participants did not significantly differ from any group (Tukey HSD tests, p < .05). Finally, there was a main effect of education, *F*(2, 940) = 3.81, *p* = .022, whereby participants with high-school degrees perceived greater myside bias than those with graduate degrees, and participants with college degrees did not significantly differ from any group (Tukey HSD tests, p < .05).

In summary, the self-other asymmetry in myside bias recognition dissipated when participants predicted side-by-side their own and others’ thoughts, feelings, and behaviors in the same situations in which myside bias might occur. Participants also attributed less myside bias overall when the self was involved in the predictions (i.e., self-other condition) than when it was not (i.e., the other-other condition), with the reduction particularly salient for the self. Thus, whereas the blindness to one’s own biases is not easily overcome, the hypersensitivity to others’ biases is attenuated when people evaluate thoughts and behaviors of others in light of their own thinking processes. In addition, older, female, Caucasian, and high-school-degree participants perceived greater myside bias in both the self and others than did younger, male, Asian, and graduate-degree participants, respectively.

## General discussion

Bias and discrimination claims have heightened social conflict and political tension in our polarized society [1–4]. The present studies set out to investigate the asymmetry in people’s recognition of myside bias and social biases in self versus others in everyday situations, to examine people’s explicit beliefs about biases as an underlying mechanism for the self-other asymmetry in bias recognition, and to further test the effectiveness of asking people to make simultaneous predictions about themselves and others for reducing the self-other asymmetry. The studies further go beyond the WEIRD (Western, educated, industrialized, rich, and democratic) populations that have dominated the samples of psychological research in general and research on cognitive biases in particular [24–26], examining the effects of age, gender, ethnicity and education on the recognition of bias in self versus others. These findings provide original theoretical insights into the persistence of and remedy for the metacognitive biased thinking and have critical real-life implications.

As predicted, people viewed preexisting beliefs and opinions as playing a greater role in determining others’ than their own thoughts, feelings, and behaviors in everyday situations (Study 1). People also viewed others as more susceptible than themselves to various social biases (Study 2). This bias in bias recognition was evident regardless of age, gender, ethnicity, and education. The view of others being vulnerable and the self being immune to the biasing influences of preexisting beliefs and social stigmas is consistent with the general asymmetry in people’s perception of self and others [14–22]. It may further reflect a metacognitive bias in which people exhibit deficiency in self-awareness, self-monitoring, or self-regulation when they apply their knowledge about biases to understand others’ and their own thoughts and behaviors in everyday situations [33, 34].

Indeed, the current studies reveal the critical role of explicit beliefs about biases in underlying the biased reasoning concerning one’s own and others’ thoughts and behaviors: The more strongly people believed that biases widely existed, the more inclined they were to ascribe biases to others but not themselves. These findings suggest that the conviction that the world is generally biased and yet the self is the exception contributes to the self-other asymmetry in bias recognition. They further suggest important individual differences whereby some individuals more strongly believe that myside bias and social biases widely exist and yet convince themselves that “I’m not one of them” when making judgements about these biases in everyday situations. In comparison, individuals who held weaker beliefs about the biases attributed less bias overall and exhibited less self-other asymmetry in recognizing the biases. These findings thus provide valuable information for future focus-group interventions. They further suggest that when learning about bias, as occurs in most introductory psychology classes, students should be reminded that they are equally susceptible as others to biasing influences.

To narrow the self-other gap in bias recognition, Study 3 attempted to make participants aware that the same rule applied to all. As predicted, when people considered simultaneously their own and others’ perspectives, the self-other asymmetry in bias recognition dissipated. The findings further suggest that the diminished self-other asymmetry in bias recognition was due to a decreased bias attribution to others, instead of an increased bias attribution to the self. Rather than making people more sensitive to their own capacity for bias, the simultaneous framing seems to increase the credibility attributed to others by anchoring them to one’s own equanimity. This may suggest a normativity tendency, where “anyone who sees things as I do will see things fairly.” It is also consistent with the literature showing the pervasiveness of motivational and cognitive processes underlying favorable self-views and the blindness to one’s own shortcomings [17, 20, 21]. These results have critical implications for real-life settings, suggesting that making people aware that we are all susceptible to the same biasing influences may reduce hypervigilance to detect biases in others—especially others who disagree with us. This may, in turn, help people become more open to different views and opinions and thus promote interpersonal understanding and reduce intergroup enmity.

Notably, although people across demographic groups consistently attributed more biases to others than to themselves, intriguing demographic differences emerged in the perception of biases more generally. Older participants perceived greater myside bias in both themselves and others than did young participants, and women perceived greater myside bias than did men. Education also played a role, such that those with lower levels of education perceived greater myside bias in both themselves and others than those with higher levels of education. Among the different ethnic groups, Caucasians perceived greatest myside bias as well as social biases in both themselves and others and Asians perceived least biases, with Hispanic and African Americans falling in between. These findings, although exploratory, suggest that bias recognition is not the mere byproduct of human cognition but may be influenced by a variety of sociocultural factors. More research is called for to understand the causes of these demographic differences and develop effective interventions accordingly.

There are some important limitations to the current research. In particular, although the Beliefs about Myside Bias (BMB) scale and the Beliefs about Social Biases (BMB) scale were designed to measure participants’ explicit beliefs about respective biases in the general population, participants might answer the questions with others in mind, not necessarily themselves. This makes the findings of the relation between explicit beliefs about biases and the self-other asymmetry in bias recognition inconclusive, and the findings need to be corroborated in additional research. Furthermore, although we used indirect measures for the self-other asymmetry in bias recognition to reduce social desirability concerns, such concerns might still be present. Future research should address this issue and further compare the magnitude of the self-other asymmetry in bias recognition when direct versus indirect measures are used. In addition, although study 3 provides evidence that social perspective-taking and mindfulness may help to eliminate the self-other asymmetry in bias recognition, mindfulness was not tested. This novel finding should serve as the basis for examining in future work the possible mechanisms. Future research should also use similar paradigms as that in Study 3 to examine the reduction of self-other asymmetry in the recognition of social biases.

The present research used original methods to understand the bias in attributing biases to others versus the self. The newly developed measurements require additional tests and corroborations. Nevertheless, this research yielded critical findings of the self-other asymmetry in the recognition of myside bias and social biases, the contributing factors to the asymmetry, and a cost-efficient remedy for reducing it. In our increasingly polarized society, it is of paramount importance for researchers to identify causes to and effective interventions for various cognitive and metacognitive biases and, in so doing, to facilitate group cohesion and social harmony.

## Supporting information

S1 File Study 1 data. (JMP)

S2 File Study 2 data. (JMP)

S3 File Study 3 data. (JMP)

S4 File Materials and measures. (DOCX)

## References

[1] Ceci S. J., & Williams W. M. (2018). Who decides what is acceptable speech on campus? Why restricting free speech is not the answer. *Perspectives on Psychological Science*, 13(3), 299–323. 10.1177/1745691618767324 29716456

[2] Ditto P. H., Liu B. S., Clark C. J., Wojcik S. P., Chen E. E., Grady R. H., et al (2019). At least bias is bipartisan: A meta-analytic comparison of partisan bias in liberals and conservatives. *Perspectives on Psychological Science*, 14, 273–291. 10.1177/1745691617746796 29851554

[3] Stanovich K. E. (2017, 9 28). Were Trump voters irrational? Quillette. Retrieved from http://quillette.com/2017/09/28/trump-voters-irrational/

[4] Ford R. T. (2008). The Race Card: How Bluffing About Bias Makes Race Relations Worse. New York: Farrar, Straus and Giroux.

[5] Tuchman B. (1984). The March of Folly: From Troy to Vietnam. New York: Knopf.

[6] Lord C. G., Ross L., & Lepper M. R. (1979). Biased assimilation and attitude polarization: The effects of prior theories on subsequently considered evidence. *Journal of Personality and Social Psychology*, 37(11), 2098–2109.

[7] Mercier H. (2017). Confirmation bias—Myside bias In Pohl R. F. (Ed.), *Cognitive illusions*: *Intriguing phenomena in thinking*, *judgment and memory*., 2nd ed (pp. 99–114). New York, NY: Routledge/Taylor & Francis Group.

[8] Plous S. (1993). McGraw-Hill series in social psychology *The psychology of judgment and decision making*. New York, NY, England: Mcgraw-Hill Book Company.

[9] Stanovich K. E., & West R. F. (2008). On the relative independence of thinking biases and cognitive ability. *Journal of Personality and Social Psychology*, 94, 672–695. 10.1037/0022-3514.94.4.672 18361678

[10] Taber C. S., & Lodge M. (2006). Motivated skepticism in the evaluation of political beliefs. *American Journal of Political Science*, 50, 755–769.

[11] Wolfe C. R., & Britt M. A. (2008). The locus of the myside bias in written argumentation. *Thinking & Reasoning*, 14, 1–27.

[12] McKenzie C. R. M. (2004). Hypothesis testing and evaluation In Koehler D. J. & Harvey N. (Eds.), *Blackwell handbook of judgment & decision making* (pp. 200–219). Malden, MA:Blackwell.

[13] Stanovich K. E., West R. F., & Toplak M. E. (2013). Myside bias, rational thinking, and intelligence. *Current Directions in Psychological Science*, 22, 259–264.

[14] Dunning D., Meyerowitz J. A., & Holzberg A. D. (1989). Ambiguity and self-evaluation: The role of idiosyncratic trait definitions in self-serving assessments of ability. *Journal of Personality and Social Psychology*, 57, 1082–1090.

[15] Williams E.F., & Gilovich T. (2008). Conceptions of the self and others across time. *Personality and Social Psychology Bulletin*, 34(8), 1037–1046. 10.1177/0146167208317603 18469152

[16] O’Sullivan O. P. (2015). The neural basis of always looking on the bright side. *Dialogues in Philosophy*, *Mental and Neuro Sciences*, 8(1),11–15.

[17] Epley N., & Dunning D. (2000). Feeling “holier than thou”: Are self-serving assessments produced by errors in self- or social prediction? *Journal of Personality and Social Psychology*, 79, 861–875. 10.1037//0022-3514.79.6.861 11138757

[18] Klein N., & Epley N. (2016). Maybe holier, but definitely less evil, than you: Bounded self-righteousness in social judgment. *Journal of Personality and Social Psychology*, 110(5), 660–674. 10.1037/pspa0000050 27176771

[19] Miller D. T, & Ratner R. K. (1998). The disparity between the actual and assumed power of self-interest. *Journal of Personality and Social Psychology*, 74, 53–62 10.1037//0022-3514.74.1.53 9457775

[20] Gilovich T., & Ross L. (2016). *The wisest one in the room*: *How you can benefit from social psychology’s most powerful insights*. New York, NY: Free Press.

[21] Pronin E., Gilovich T., & Ross L. (2004). Objectivity in the eye of the beholder: Divergent perceptions of bias in self versus others. *Psychological Review*, 111, 781–799. 10.1037/0033-295X.111.3.781 15250784

[22] Ehrlinger J., Gilovich T., & Ross L. (2005). Peering into the bias blind spot: People’s assessments of bias in themselves and others. *Personality and Social Psychology Bulletin*, 31, 680–692. 10.1177/0146167204271570 15802662

[23] Pronin E., & Kugler M. B. (2007). Valuing thoughts, ignoring behavior: The introspection illusion as a source of the bias blind spot. *Journal of Experimental Social Psychology*, 43(4), 565–578.

[24] Rad M. S., Martingano A. J., & Ginges J. (2018). Toward a psychology of *Homo sapiens*: Making psychological science more representative of the human population. *Proceedings of the National Academic of Sciences*, 115(45), 11401–11405. 10.1073/pnas.1721165115 30397114 PMC6233089

[25] Wang Q. (2016). Why should we all be cultural psychologists? Lessons from the study of social cognition. *Perspectives on Psychological Science*, 11(5), 583–596. 10.1177/1745691616645552 27694456 PMC5119767

[26] Wang Q. (2021). The cultural foundation of human memory. *Annual review of Psychology*, 72 10.1146/annurev-psych-070920-023638 32928062

[27] Fisher R. J. (1993). Social Desirability Bias and the Validity of Indirect Questioning, *Journal of Consumer Research*, (20)2, 303–315. 10.1086/209351

[28] Apperly I. (2010). *Mindreaders*: *the cognitive basis of “theory of mind”*. London: Psychology Press 10.4324/9780203833926

[29] Davis M. (1980). A multidimensional approach to individual differences in empathy. *Catalogue of Selected Documents in Psychology*, 10, 85.

[30] Wolgast A., Tandler N., Harrison L. & Umlauft S. (2019). Adults’ dispositional and situational perspective-taking: A systematic review. *Educational Psychology Review*. 10.1007/s10648-018-9458-2 30930595 PMC6407857

[31] Buhrmester M., Kwang T., & Gosling S. D. (2011). Amazon's Mechanical Turk: A new source of inexpensive, yet high-quality, data? *Perspectives on Psychological Science*, 6(1), 3–5 26162106 10.1177/1745691610393980

[32] Peer E., Vosgerau J., Acquisti A. (2014). Reputation as a sufficient condition for data quality on Amazon mechanical Turk. *Behavioral Research Methods*, 46(4),1023–1031. 10.3758/s13428-013-0434-y 24356996

[33] Briñol P. & DeMarree K. G. (2012) (Eds). Social Metacognition. New York, NY: Psychology Press 10.4324/9780203865989

[34] Yzerbyt V. Y., Schadron G., Leyens J-P, & Rocher S. (1994). Social judgeability: The impact of meta-informational cues on the use of stereotypes". *Journal of Personality and Social Psychology*, 66, 48–55. 10.1037/0022-3514.66.1.48

