# Supplementary material for: Bias in bias recognition: People view others but not themselves as biased by preexisting beliefs and social stigmas
Source: PLoS One. 2020 Oct 9;15(10):e0240232. doi: 10.1371/journal.pone.0240232 (PMC7546453; doi:10.1371/journal.pone.0240232)
Supplement: S4 File — (DOCX) [file pone.0240232.s004.docx]

Materials and Measures

**Study 1**

**Scenarios**

1. Jessica is (You are) interested in celebrity fashion. Jessica (You) heard that Princess M was attending a charity event wearing a white dress. Jessica (You) then saw a picture of Princess M at the event, wearing a whitish-pinkish dress (a color that can be seen as either white or pink). What color would Jessica (you) remember that Princess M wore at the event?

2. John (You) and Eric are friends who spend a lot of time together. Deep down, John thinks (you think) that Eric is a boring person. Two weeks ago on Saturday, John (you) went to Eric’s party and had a great time. When remembering back about Eric’s party, how would John (you) feel about it?

3. Jack (You) signed up to play soccer for the first time and needs (need) to buy soccer cleats. Jack (You) saw his (your) least favorite basketball team wear Adidas shorts in a recent game. When Jack gets (you get) to the store, there are only two brands of cleats on sale: Nike and Adidas. Which is the likely brand of cleats that Jack (you) would buy?

4. Nancy is (You are) a professor evaluating a job candidate, Bill, for her (your) department. Nancy knows (You know) Bill personally and really likes (like) him. Bill has published on average 3 articles a year, which is about the same as the average number of publications by the faculty in the department. How would Nancy (you) evaluate Bill’s performance?

5. Janice is (You are) hungry and thinking about what to eat for lunch while walking to Koko’s, a small café on campus. Janice has (You have) heard that Koko’s budae-jjigae is overpriced and salty. When Janice gets (you get) to Koko’s, budae-jjigae is the only thing available to order. How would Janice (you) feel about her (your) lunch at Koko’s after eating?

6. Erica (You) took a Calculus class with Professor Phalange because her (your) friends had highly recommended it. The class turned out to be very difficult and no one did well. Erica (You) received a B-, an average grade for the class. When filling out the end of the year course evaluations, how would Erica (you) rate Professor Phalange’s class?

7. Justin (You) went to the movies to watch La La Land, a musical comedy-drama film starring Emma Stone and Ryan Gosling. Justin (You) generally dislikes (dislike) musicals and Broadway shows, but he (you) enjoyed the movie and had a good time. A few days later, a friend asks for his (your) recommendation about the movie. How many stars would Justin (you) give La La Land?

8. Steve (You) and Sarah work in the same company. Steve dislikes (You dislike) Sarah and thinks (think) she is mean. People in the company were asked to donate money for a colleague whose house was recently burned down. Sarah put in $50, which was about the average amount of what people donated. What would Steve (you) think about the amount of Sarah’s donation?

9. Sally knows (You know) for sure that Ucro is a precious metal used for high-end jewelry. Sally sees (You see) someone selling an Ucro necklace online for $50. How would Sally (you) think of the necklace?

10. Sylvia was (You were) at an ice hockey game rooting for the home team. The away team and her (your) team each scored two goals and tied the match. How would Sylvia (you) rate her (your) team’s performance in the game?

11. Brandon (You) learned in an archeology class that Anker is a type of a bronze spearhead that was used for fighting thousands of years ago. Brandon meets (You meet) a person claiming to have found Ankers in areas where ancient farming took place. How would Brandon (you) think of the claim?

12. Jacob (You) actively endorses (endorse) Dooyu, a prominent soy milk company, for their donations to various humanitarian groups. At lunch, Jacob is (you are) given two soy milk products: one from Dooyu and the other from Rego, a rival soy milk company that produces similar tasting soy milk. Which soy milk would Jacob (you) prefer after tasting?

13. Casey believes (You believe) the claim that eating apples everyday helps the body stay healthy is a marketing scheme to sell apples. In a recent health report, it says that eating 3 apples a day is highly recommended for good health. How likely would Casey (you) follow this recommendation?

14. Sam is (You are) looking to buy one of the new ASO computers that are considered by experts to be the most advanced computers on the market. At a social media site, Sam reads (you read) that according to a technician at Frogle Inc., the ASO computers at the corporate office are unable to run simple programs. Would Sam (you) still buy an ASO computer?

15. Carol thinks (You think) that fiction books are boring. A fiction book was published a year ago, which received outstanding reviews and multiple awards shortly after it came out. Carol (You) read the book out of curiosity and sort of liked it. When remembering back about the book, how would Carol (you) think of the book?

16. Ode is (You are) looking for a babysitter. Jenny and Tina have both made excellent impressions and both have great references. Jenny is recommended by one of Ode’s (your) friends. Tina has worked for families whom Ode is (you are) not acquainted with. Which person would Ode (you) choose as the new babysitter?

**Beliefs about Myside Bias Scale (BMB)**

Please indicate how strongly you agree or disagree with each of the following statements.

0---------1---------2----------3----------4----------5----------6----------7----------8----------9---------10

Strongly disagree Strongly agree

1. People are generally biased in their thinking.
2. People see what they want to see.
3. People are open to views different from their own.
4. People find excuses to refute a position they disagree with.
5. People choose to believe what they want to believe.
6. People’s judgments are not clouded by their prior beliefs.
7. People tend to remember things consistent with their views.
8. People often accept things that disprove their beliefs.
9. People seek out information to defend their positions.
10. People are generally impartial when making judgments.

**Study 2**

**Scenarios**

**Sexism**

1. Leslie chairs (you chair) the search committee to hire a lecturer for the psychology department. The finalists are Sara and Chris, who are equally qualified in every aspect. Which candidate are Leslie (you) and the committee likely to hire?

Assign a percentage to each candidate (Total = 100%):

Sara ___________________ %

Chris ___________________%

2. Alex is (you are) the director on a board that decides funding for student organizations. There is limited funding this year that can only support either the men’s soccer team or the women’s soccer team. Which team are Alex (you) and the board likely to fund?

Assign a percentage to each team (Total = 100%):

Men’s soccer team ___________________ %

Women’s soccer team _________________%

3. Noel (you), the head of the design department, is (are) to recommend one of the members for a prestigious fellowship. Two people are particularly deserving, Jane and Adam. Which member is Noel (are you) likely to recommend?

Assign a percentage to each member (Total = 100%):

Jane ___________________ %

Adam __________________%

4. Sterling is (you are) looking around for a neurologist for an aging parent. Two doctors are highly recommended by others, Tom and Emily. Which doctor is Sterling (are you) likely to choose?

Assign a percentage to each doctor (Total = 100%):

Tom ___________________ %

Emily __________________%

**Ageism**

1. Lee is (you are) the recruitment manager for a tech company. Two applicants, 25-year-old Adam and 55-year-old Seth, are in the final round for the programmer position. Which programmer is Lee (are you) likely to choose?

Assign a percentage to each applicant (Total = 100%):

Adam ___________________ %

Seth ____________________%

2. Bailey is (you are) the director of a dance troupe. Two dancers have made it to the final round of auditions for the lead dancer: 38-year-old Linsey and 21-year-old Sarah. Which dancer is Bailey (are you) likely to choose?

Assign a percentage to each dancer (Total = 100%):

Linsey ___________________ %

Sarah ____________________%

3. River is (you are) the owner of a family restaurant. Two people, 27-year-old Michael and 53-year-old Ross, applied for the job of serving food to the customers. Which applicant is River (are you) likely to choose?

Assign a percentage to each applicant (Total = 100%):

Michael ___________________ %

Ross ______________________%

4. Skyler is (you are) a client looking for an attorney for a court hearing. The possible candidates have been narrowed down to two equally skilled attorneys: 65-year-old Bridget and 42-year-old Melissa. Which attorney is Skyler (are you) likely to choose?

Assign a percentage to each attorney (Total = 100%):

Bridget ___________________ %

Melissa ___________________%

**Racism**

1. Robin is (you are) a university dean looking to hire a senior associate dean in charge of important research and outreach activities in the college. The decision has been narrowed down to two finalists: Juan Martinez and William Silbey. Which candidate is Robin (are you) likely to choose?

Assign a percentage to each candidate (Total = 100%):

Juan Martinez ___________________ %

William Silbey __________________%

2. Wynne is (you are) looking to hire a limo driver. The limo company has suggested two excellent drivers who have never been in an accident before: Kelly Smith and Lakisha Lacks. Which driver is Wynne (are you) likely to choose?

Assign a percentage to each driver (Total = 100%):

Kelly Smith ___________________ %

Lakisha Lacks _________________%

3. Caden (you), a dentist at a dental clinic, is (are) searching for an assistant. Two applicants, Ximena Washington and Ashely Johnson, with similar background and experiences, have applied for the position. Which dental assistant is Caden (are you) likely to choose?

Assign a percentage to each applicant (Total = 100%):

Ximena Washington_________________%

Ashely Johnson ____________________ %

4. Justice is (you are) a master French Cuisine chef looking to hire a sous chef. Through a cooking demonstration, Billy Brown and Mateo Begay were determined to be equally talented. Which chef is Justice (are you) likely to choose as the new sous chef?

Assign a percentage to each chef (Total = 100%):

Billy Brown ___________________ %

Mateo Begay __________________%

**Classism**

1. Beck is (you are) the owner of an apartment building. There are two tenants, John – a college professor, and Max – a plumber, who are equally interested and willing to pay the full price for the last apartment within the building. Which tenant is Beck (are you) likely to choose?

Assign a percentage to each tenant (Total = 100%):

John _________________%

Max _________________%

2. Caro works (you work) at a restaurant as a part-timer. A construction worker and a lawyer are both there alone and have been waiting in line for 20 minutes to get seated. Which customer is Caro (are you) likely to seat first?

Assign a percentage to each customer (Total = 100%):

Construction Worker _________________%

Lawyer ____________________________%

3. Lior is (you are) the head coach of a university tennis team. Two equally qualified players are fighting for the captain position: Lisa from Mckeesport, Pennsylvania, and Jane from the upper East side of New York City. Which player is Lior (are you) likely to choose as the new tennis team captain?

Assign a percentage to each player (Total = 100%):

Lisa _________________%

Jane _________________%

4. Ode is (you are) looking for a babysitter. Jed, a college graduate, and Tim, a high school drop-out, have both made excellent impressions and are similar in personality. Which person is Ode (are you) likely to choose as the new babysitter?

Assign a percentage to each candidate (Total = 100%):

Jed _________________%

Tim _________________%

**Beliefs about Social Biases Scale (BSB)**

Please indicate how strongly you agree or disagree with each of the following statements.

0---------1---------2----------3----------4----------5----------6----------7----------8----------9---------10

Strongly disagree Strongly agree

1. Most people have the tendency of being racist.
2. People are generally ageist.
3. People are often biased against those from lower social-economic status.
4. Many people are sexist.

**Study 3**

**Scenarios**

1. Jessica (You) and Emily are both interested in celebrity fashion. They (You) both heard that Princess M was attending a charity event.

Emily heard that Princess M was wearing Pink to the event.

Jessica (You) heard that Princess M was wearing White to the event.

They (You) then both saw a picture of Princess M at the event, wearing a whitish-pinkish dress (a color that can be seen as either white or pink).

What color would Jessica (you) and Emily remember that Princess M wore at the event?

White somewhere in between Pink

Jessica (You)

|________|_______|________|________|________|________|________|________|

-4 -3 -2 -1 0 1 2 3 4

Emily

|________|_______|________|________|________|________|________|________|

-4 -3 -2 -1 0 1 2 3 4

2. John (You) and Adam are both friends with Eric. The three of them (you) spend a lot of time together.

Deep down, Adam thinks that Eric is a boring person.

In general, John thinks (you think) that Eric is a fun-loving guy.

John (You) and Adam went to Eric’s party on Saturday two weeks ago and had a great time.

When remembering back about Eric’s party, how would John (you) and Adam feel about it?

Fun somewhere in between Boring

John (You)

|________|_______|________|________|________|________|________|________|

-4 -3 -2 -1 0 1 2 3 4

Adam

|________|_______|________|________|________|________|________|________|

-4 -3 -2 -1 0 1 2 3 4

3. Jack (You) and Nick signed up to play soccer for the first time and need to buy soccer cleats.

Jack (You) saw his (your) least favorite basketball team wear Adidas shorts in a recent game.

Nick recently watched a cool commercial of Adidas T-shirts.

When they (you and Nick) get to the store, there are only two brands of cleats on sale: Nike and Adidas.

Which is the likely brand of cleats that Jack (you) and Nick would buy?

Adidas somewhere in between Nike

Jack (You)

|________|_______|________|________|________|________|________|________|

-4 -3 -2 -1 0 1 2 3 4

Nick

|________|_______|________|________|________|________|________|________|

-4 -3 -2 -1 0 1 2 3 4

4. Nancy (You) and Linda are both professors in the same department. They (You two) are evaluating a job candidate, Bill.

Nancy knows (You know) Bill personally and really likes (like) him.

Linda knows about Bill’s work and doesn’t think highly of it.

Bill has published on average 3 articles a year, which is about the same as the average number of publications by the faculty in the department.

How would Nancy (you) and Linda evaluate Bill’s performance?

Unproductive somewhere in between Productive

Nancy (You)

|________|_______|________|________|________|________|________|________|

-4 -3 -2 -1 0 1 2 3 4

Linda

|________|_______|________|________|________|________|________|________|

-4 -3 -2 -1 0 1 2 3 4

Scenario 5

5. Janice (You) and Judy are hungry and thinking about what to eat for lunch. They (You two) walk to Koko’s, a small café on campus.

Judy has heard that Koko’s budae-jjigae is delicious.

Janice has (You have) heard that Koko’s budae-jjigae is overpriced and salty.

When they (you two) get to Koko’s, budae-jjigae is the only thing available to order.

How would Janice (you) and Judy feel about their (your) lunch at Koko’s after they (you) eat?

Terrible somewhere in between Delicious

Janice (You)

|________|_______|________|________|________|________|________|________|

-4 -3 -2 -1 0 1 2 3 4

Judy

|________|_______|________|________|________|________|________|________|

-4 -3 -2 -1 0 1 2 3 4

Poor somewhere in between Excellent

Erica (You)

|________|_______|________|________|________|________|________|________|

-4 -3 -2 -1 0 1 2 3 4

Dara

|________|_______|________|________|________|________|________|________|

-4 -3 -2 -1 0 1 2 3 4

When filling out the end of the year course evaluations, how would Erica (you) and Dara rate Professor Phalange’s class?

The class turned out to be very difficult and no one did well. Both Erica (you) and Dara received a B-.

6. Erica (You) and Dara both took the Calculus class with Professor Phalange.

Dara had heard bad things about the class, but had to take it to fulfill a requirement.

Erica (You) took this class because her (your) friends had highly recommended it.

7. Justin (You) and Brian went to the movies to watch La La Land, a musical comedy-drama film starring Emma Stone and Ryan Gosling.

1 Star somewhere in between 5 Star

Justin (You)

|________|_______|________|________|________|________|________|________|

-4 -3 -2 -1 0 1 2 3 4

Brian

|________|_______|________|________|________|________|________|________|

-4 -3 -2 -1 0 1 2 3 4

How many stars would Justin (you) and Brian give La La Land?

They (You) both enjoyed the movie and had a great time. A few days later, a friend asks for their (your and Brian’s) recommendation about the movie.

Brian loves Emma Stone and Ryan Gosling too, but hates musicals and Broadway shows.

Justin loves (You love) Emma Stone and Ryan Gosling, as well as musicals and Broadway shows.

Steve dislikes (You dislike) Sarah and thinks (think) she is mean.

Bob likes Sarah and thinks she is nice.

8. Steve (You), Bob, and Sarah work in the same company.

What would Steve (you) and Bob think about the amount of Sarah’s donation?

People in the company were asked to donate money for a colleague whose house was recently burned down. Sarah put in $50.

Very Meager somewhere in between Very Generous

Steve (You)

|________|_______|________|________|________|________|________|________|

-4 -3 -2 -1 0 1 2 3 4

Bob

|________|_______|________|________|________|________|________|________|

-4 -3 -2 -1 0 1 2 3 4
